# Gender dysphoria: Psychological impact and social repercussions

**DOI:** 10.1192/j.eurpsy.2023.2342

**Published:** 2023-07-19

**Authors:** I. Belabbes, M. Chtibi, K. Douk, H. Kisra

**Affiliations:** Arrazi hospital, Sale, Morocco

## Abstract

**Introduction:**

Gender dysphoria is defined as a multisystemic medical condition in which a person has a marked mismatch between their biological sex and the gender with which they identify.

**Objectives:**

To highlight the psychological impact and social repercussions of gender dysphoria and to discuss the different aspects of management aimed at optimising a better quality of life for these patients.

**Methods:**

We describe the clinical cases of 5 patients followed at the child psychiatry department and the adolescent diagnostic centre of agdal, who were diagnosed with gender dysphoria.

**Results:**

Clinical vignette:

- A.B: 15-year-old patient, followed in our training for a recurrent depressive disorder comorbid with borderline personality and gender dysphoria. This patient is a victim of school bullying altering his psychosocial functioning and generating thoughts of death.
- H.A: 16 year old patient, followed in our training for gender dysphoria comorbid with adrenal hyperplasia, indicating feminization surgery.
- I.D: 17 year old female patient, victim of sexual assault, admitted to our training for suicide attempt. She presents a gender dysphoria, comorbid with a borderline personality.
- C.G: 22 year old patient, followed in our training for gender dysphoria comorbid with a panic disorder. She is a patient describing an anxious experience with dysthymia.
- L.K: 23-year-old patient, followed in our training for gender dysphoria. He is a patient who would have been a victim of verbal and physical aggression generating a post-traumatic stress disorder having had a significant impact on his socio-professional life.

**Conclusions:**

Primary care physicians need to be aware of gender-related disorders and the importance of early recognition of these emerging disorders. A multidisciplinary approach is needed to manage these disorders.

**Disclosure of Interest:**

None Declared

